# Pedants of Superlative Calibre

**Published:** 1892-09-10

**Authors:** 


### PEDANTS OF SUPERLATIVE CALIBRE.

The *Standard* the other day surpassed itself in the art of phrase making. Professor Max Muller, whom we all venerate, had for once forgotten his common sense, and from the chair of the Oriental Congress had laid down the astoundingly stupid rule that nobody ought to be called an Orientalist unless he had translated some original work of ancient Oriental literature. According to this, all the thoughtful Europeans who have spent their whole lives in India, and who have devoted their time to the completest possible study of living India, and of the Indian literature of all past times, are not worthy to be called Orientalists, whilst some competition Wallah, who has passed well in Hindustanee and has had a little spare time to translate the least important of ancient Eastern writings, is properly called an Orientalist. This is absurdity of the very sublimest order. It is the same as saying that a Board School master who may have translated a physiological treatise from the French is a physiologist, whilst the physiological professor at a hospital who has never done so is not a physiologist. Truly, even specialism never took up a sillier position than this! Well may the *Standard* speak of "Pedants of Superlative Calibre."

